# Evaluation and Comparison of Facial Appearance Using the Golden Ratio: An Anthropometric Study in Preschool and School-Going Children of Santhal Tribe in West Bengal

**DOI:** 10.7759/cureus.53200

**Published:** 2024-01-29

**Authors:** Vipin Ahuja, Annapurna Ahuja, Nilima R Thosar

**Affiliations:** 1 Pediatric and Preventive Dentistry, Hazaribag College of Dental Sciences and Hospital, Hazaribag, IND; 2 Periodontics and Implant Dentistry, Hazaribag College of Dental Sciences and Hospital, Hazaribag, IND; 3 Pediatric and Preventive Dentistry, Sharad Pawar Dental College and Hospital, Datta Meghe Institute of Higher Education and Research, Wardha, IND

**Keywords:** beauty, mixed dentition, primary teeth, anthropometry, divine proportion, golden ratio

## Abstract

Introduction

Golden ratio and beauty are two inseparable sides of the same coin and have been studied for centuries by the Greeks. This divine ratio is defined as an invincible parameter in aesthetic dentistry to measure looks, symmetry, and balance. Being beautiful and handsome also boosts confidence in today’s children and therefore is a top priority for young growing kids. However, there is no study done to define facial measurements based on the golden ratio in preschool and school-going children that can succor aesthetics in formative years. The purpose of this research was to evaluate facial proportions in the vertical dimension, quantify them in reference to the golden ratio, and analyze the association with gender among preschool and school-going children of the Santhal tribe in the Bankura district.

Materials and methods

A total of 399 subjects, 198 children of 3-5 years with primary teeth and 201 children of 6-12 years with mixed dentition, were selected from schools in villages of Bankura district, West Bengal, India. The subjects were made to relax in a sitting position and a digital vernier caliper was used to record the following vertical facial parameters: total facial height (TFH), trichion-gnathion distance (Tr-Gn), and subnasale-gnathion distance (Sn-Gn). The total facial height was correlated with sexual dimorphism and dentition. The ratio of Tr-Sn/Sn-Gn was calculated and compared with the golden ratio. The ratio was classified as normal (if it was between 1.6 and 1.699, i.e., normal to the golden ratio value), long (if it was more than 1.699, i.e., more than the golden ratio value), or short (if it was below 1.6, i.e., less than the golden ratio value). This facial analysis based on the golden ratio was correlated to sexual dimorphism and dentition. The data were recorded, compared with gender, and statistically analyzed using the unpaired t-test and Chi-square test.

Results

The total facial height was larger in males than females in both primary and mixed dentition; however, the value was highly significant in primary dentition. Tr-Sn/Sn-Gn ratios were lower in the long facial category in both males and females in both preschool and school-going children. The values were statistically significant in these ratios for both dentitions.

Conclusion

The majority of children in the Santhal tribe of Bankura in West Bengal did not conform to the golden ratio and showed long faces. There was a significant association of facial features with sexual dimorphism.

Clinical significance

The early prediction of facial features in children and its confirmation with the established golden ratio can be considered an imperative parameter to comprehend facial aesthetics and symmetry.

## Introduction

The golden ratio is a primitive yet significant segment of aesthetic science. It is the proportion used to qualify and quantify beauty in Greeks and is termed the divine proportion. Phidia, a Greek sculptor had widely incorporated this golden ratio in his sculptures. As a tribute to his works, the term ‘Phi’ was used to denote this ratio, and the symbol was invented by Mark Barr. The universe is full of examples of the golden ratio such as animal or human skeletons, branches of plants, hands, fingers, etc., and other man-made things like the Mona Lisa painting and several iconic buildings, and this divine ratio has been considered for centuries. Greek mathematician Euclid first defined the golden ratio and later Filius Bonacci gave a precise numerical value of 1 to 1.61803399. The literature is full of studies where the golden ratio is considered an ideal value to define symmetry and balance for the human face and body [1-3]. One prominent example is the ‘Bust of Nefertiti’, a limestone sculpture of Queen Nefertiti, an iconic beauty known for her beautiful facial features and proportions to an extent that even after 3,500 years plastic surgeons consider her an unparalleled beauty with standardized lateral and frontal features. A study has quoted that the naso-oral proportions and lip features of the sculpture of Nefertiti are in approximation to the golden ratio. This divine ratio or proportion is also highlighted as a reliable marker for quantifying the beauty and attractiveness of the face [4,5].

The golden proportion is an elusive formula to quantify harmony and balance in dental relationships and has been quoted in literature with lucid examples. The arrangement of maxillary anterior teeth with respect to mandibular anterior teeth and maxillary posterior teeth, proportions of anterior teeth, and inter-arch relationships can be truly correlated with the golden ratio. One of the prominent illustrations is the width of the maxillary incisors. The width of the maxillary central incisor is 1.6 times larger than the width of the maxillary lateral incisor, and similarly, the width of a maxillary lateral incisor is 1.6 times larger than the width of the visible part of the maxillary canine. Another example is the width-to-height ratio of incisors; it is documented by Dr. Stephen Marquardt that the combined width of two incisors crowns to the height of crown ratio is 1.6 to 1, which is the golden ratio [6].

Beauty and health are interrelated and this biological connection has been documented. Beauty if described with reference to the divine proportion as ideal or near to ideal is healthy. As per the golden ratio, mesocephalic faces being closer to the ratio are healthier. Conversely, brachycephalic or short faces are linked with temporomandibular joint (TMJ) and myofascial disorders, while dolichocephalic or long faces are linked to upper airway problems and mouth breathing issues. As a point of significance, it is not only about being beautiful but also about being healthy if features are close to the golden ratio. In this era of facial looks and aesthetics, where beauty often takes center stage in people's priorities, it is not only imperative to comprehend it for adults but also for children. One study has compared longitudinally three ages of the same individuals in childhood, youth, and mid-age and found that facial features show minimal changes with age and so can be studied at an early age [7].

But to our surprise, very few handfuls of research have been considered in the past to evaluate and study facial and dental patterns in young children; therefore, this study was undertaken to quantify facial features in preschool children of 3-5 years of age with primary dentition and school-going children of 6-12 years with mixed dentition and also the correlation with gender was studied. And as far as our literature search goes, this is the first study where average facial values were recorded for both primary and mixed dentition comprehensively and compared with the golden ratio in children of the Santhal tribe in Bankura.

## Materials and methods

A total of 399 subjects, 198 children of 3-5 years with primary teeth and 201 children of 6-12 years with mixed dentition, were selected from schools in villages of Bankura district, West Bengal, India. Sample size calculation was done using the software G*Power v3.1.9.4 (Heinrich-Heine-Universität Düsseldorf, Germany). Based on the reference article for unpaired t-test, the effect size was kept at 0.41, alpha error 5%, and power of the study 80%. The total minimum sample size for the study required was 180, and 95 was the minimum number for both groups individually. For the Chi-square test, the total minimum sample size required was 88.

The study area was the private schools under the care of Shaimayitah Math in Ranabahai village of Bankura district. A well-trained, single examiner from the Department of Pediatric and Preventive Dentistry was deployed for this study. A stratified random sampling technique was used to select the sample. Written consent from parents was taken and ethical committee approval was sought prior to the study from the Institutional Ethical Committee (IEC) of Hazaribag College of Dental Sciences and Hospital, Hazaribag.

The inclusion criteria were: children from the Santhal tribe of Bengali ethnic origin, children with primary or mixed dentition, preschool children with all primary teeth, and school-going children with mixed dentition below 12 years of age without any congenital anomaly or absence of teeth. The exclusion criteria were: children with significant asymmetry of the face with or without known etiology, children with facial paralysis, or children with a history of facial surgery or reconstruction.

**Figure 1:**
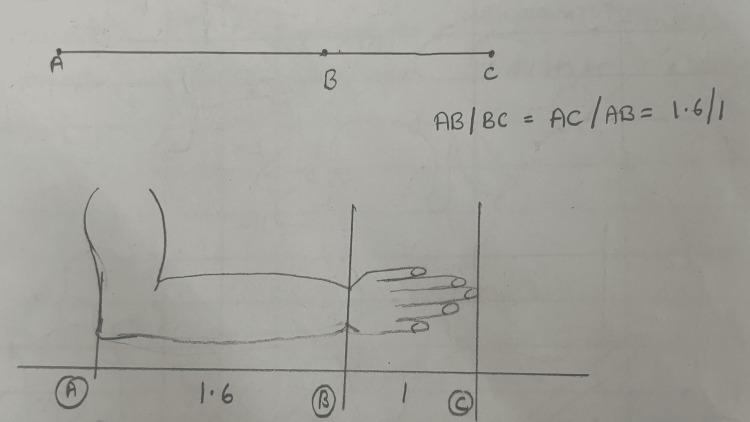
Calculation of the golden ratio

The calculation of the golden ratio was done in a standardized way. A geometric way of calculating the golden ratio is shown (Figure 1). A straight line is drawn from point A to point C and point B divides the line into two parts, part AB and part BC. The golden ratio is 1.6:1 and is calculated as AB/BC=AC/AB. In simple terms, the AB to BC ratio falls at 1.6:1 if it coincides with the golden ratio. This ratio can be applied to body parts like arms; for instance, if the upper arm is divided at the wrist then the ratio between elbow-to-wrist distance and wrist-to-fingers endpoint distance shall fall in the golden ratio [8,9].

**Figure 2:**
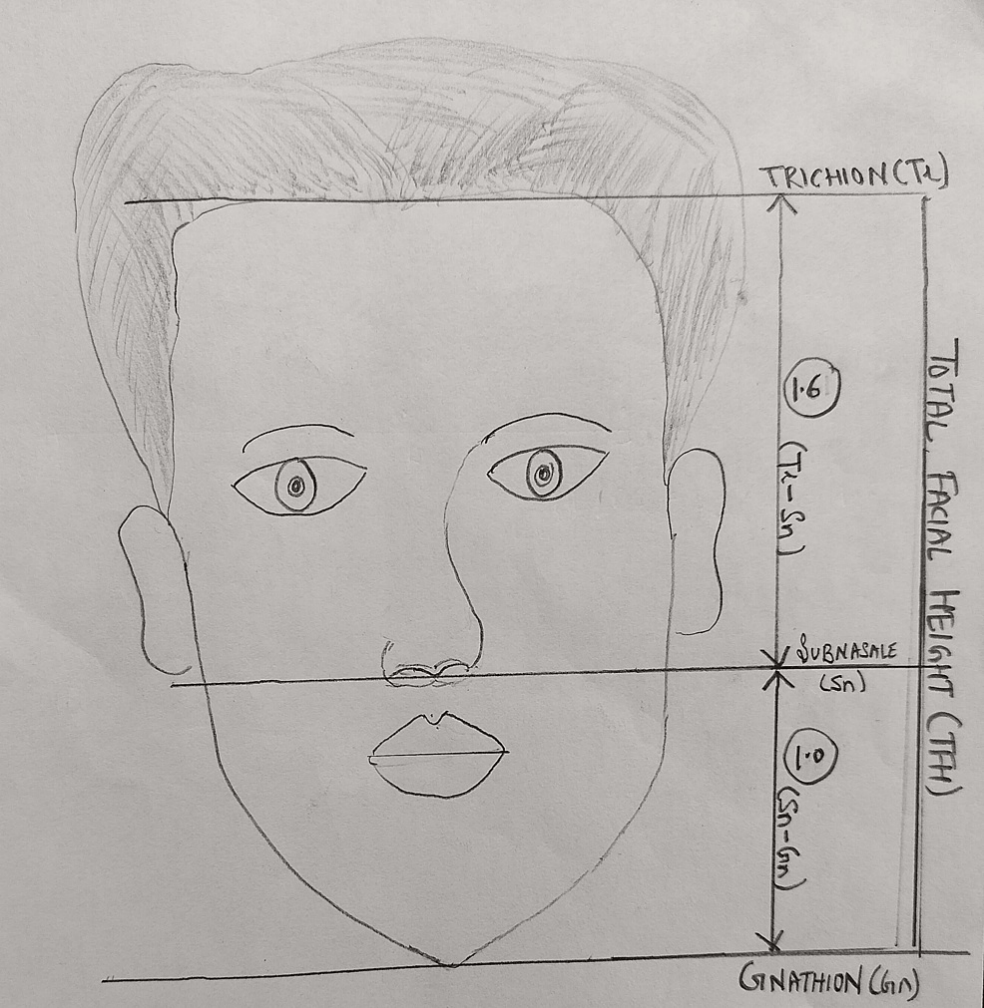
Anatomical landmarks and facial parameters used to compare with the golden ratio

In our methodology, direct measurement anthropometry with a digital vernier caliper was used instead of photographic anthropometric analysis. The reason was a surplus of studies that prove the reliability of direct anthropometry and a study by Franke-Gromberg C et al. wherein it was inferred that direct measurement is more reliable and that in photographic cephalometry images are 7.6% shorter than normal. The subjects were made to relax in a sitting position with an upright and unsupported head. They were asked to close their mouth in a comfortable position. The subjects were instructed to swallow and relax without separating the lips [10,11]. Anatomical landmarks and parameters used in the study to compare facial ratios with the golden ratio are shown (Figure 2). The following anatomical landmarks were marked with a marker on the face to record the parameters trichion, subnasale, and gnathion in a standardized manner: trichion (Tr) is marked as a point at the anterior hairline in the midline, subnasale (Sn) is marked as a point at the junction of nasal septum and upper lip, and gnathion (Gn) is marked as a point at the most inferior soft-tissue point on chin.

A digital vernier caliper was used to record the following vertical facial parameters: total facial height (TFH) as trichion-gnathion distance, trichion-subnasale distance (Tr-Sn), and subnasale-gnathion distance (Sn-Gn). The Tr-Sn/Sn-Gn ratio was calculated and evaluated in reference to the golden ratio and was classified as follows [8]:

A. Normal (normal to golden ratio, meaning value between 1.6-1.699)

B. Long (more than golden ratio, meaning value more than 1.699)

C. Short (less than golden ratio, meaning value less than 1.6)

Three readings of distance measurements were recorded and the mode of these readings was noted as the final reading to enable standardization in measurements. Data were recorded and compared with gender and statistically analyzed using Statistical Package of Social Sciences software (SPSS version 19.0; Chicago Inc., USA). The numerical values were calculated as mean, standard deviation, and 95% confidence interval using an unpaired t-test, and a Chi-square test was applied for the comparison of ratios.

## Results

Among the total 399 children, 198 children of 3-5 years with primary teeth (102 males and 96 females) and 201 children of 6-12 years with mixed dentition (94 males and 107 females) from the Santhal tribe participated in the study.

Sex-wise distribution of total facial height in preschool and school-going children of the Santhal tribe of West Bengal is shown in Table 1. It is revealed in the present study of 399 children that total facial height is greater in males than females in both preschool and school-going children, and it is recorded in centimeters (cm). The mean total facial height recorded is 14.65 cm in boys and 14.23 cm in girls with primary dentition, and the difference is statistically significant. The mean total facial height recorded is 15.75 cm for boys and 15.68 cm for girls with mixed dentition, but the difference is not statistically significant.

**Table 1:** Total facial height in preschool and school-going children and its association with sexual dimorphism Tr-Gn: Trichion-gnathion distance; SD: Standard deviation; M: Male, F: Female

Total facial height in preschool children (Tr-Gn)							
Parameter	Gender	Mean	SD	95% Confidence Interval		M vs F	
				Lower	Upper	t	P
Total length of face (cm)	Male	14.65	1.09	12.47	16.84	2.77	0.006
	Female	14.23	1.08	12.07	16.38		
Total facial height in school-going children (Tr-Gn)							
Parameter	Gender	Mean	SD	95% Confidence Interval		M vs F	
				Lower	Upper	t	P
Total length of face (cm)	Male	15.75	0.18	15.39	16.11	0.45	0.65
	Female	15.68	1.04	13.60	17.76		

The Tr-Sn/Sn-Gn ratio and its correlation with sex in preschool and school-going children of the Santhal tribe of West Bengal is shown in Table 2. It is revealed in the present study of 399 children that the Tr-Sn/Sn-Gn ratio is lower in males than females in both preschool and school-going children. The mean ratio in boys with primary dentition is 1.93 as compared to 1.97 in girls, and the difference is statistically non-significant. The mean ratio in boys with mixed dentition is 1.86 as compared to 1.90 in girls, but the difference is not statistically significant.

**Table 2:** Tr-Sn/Sn-Gn ratio and sexual dimorphism in preschool and school-going children Tr-Gn: Trichion-gnathion distance; Sn-Gn: Subnasale-gnathion distance; SD: Standard deviation; M: Male, F: Female

Tr-Sn/Sn-Gn ratio and sexual dimorphism in preschool children with primary dentition							
Parameter	Gender	Mean	SD	95% Confidence Interval		M vs F	
				Lower	Upper	t	P
Preschool children with primary dentition	Male	1.93	0.15	1.63	2.24	-1.76	0.08
	Female	1.97	0.17	1.63	2.32		
Tr-Sn/Sn-Gn and sexual dimorphism in school-going children with mixed dentition							
Parameter	Gender	Mean	SD	95% Confidence Interval		M vs F	
				Lower	Upper	t	P
School-going children with mixed dentition	Male	1.86	0.19	1.48	2.24	1.52	0.13, NS
	Female	1.90	0.18	1.54	2.26		

The Tr-Sn/Sn-Gn ratio with reference to the golden ratio in preschool children of the Santhal tribe of West Bengal is shown in Table 3. It is revealed in the present study that in preschool children with primary dentition majority of the population falls in the long face category. 6.9% of boys and 0% of girls fall in the golden ratio, whereas only 1.9% of boys and 3.1% of girls fall in the short face category. The majority, 91.2% of boys and 96.9% of girls fall in the long face category.

**Table 3:** Tr-Sn/Sn-Gn ratio evaluation with reference to the golden ratio in preschool children Tr-Gn: Trichion-gnathion distance; Sn-Gn: Subnasale-gnathion distance

Tr-Sn/Sn-Gn ratio evaluation with reference to the golden ratio in preschool children						
Preschool children with primary dentition	Male		Female		Total	
	No.	%	No.	%	No.	%
Normal	7	6.9	0	0	7	3.6
Long	93	91.2	93	96.9	186	93.9
Short	2	1.9	3	3.1	5	2.5
Total	102	100.0	96	100.0	198	100.0
X² = 7.02, P = 0.03, S						

The Tr-Sn/Sn-Gn ratio with reference to the golden ratio in school-going children of the Santhal tribe of West Bengal is shown in Table 4. In school-going children with mixed dentition, 6.4% of boys and 9.4% of girls fall in the golden ratio (1.6-1.699), whereas only 5.3% of boys and 3.7% of girls fall in the short face category (less than 1.6). The majority, 88.3% of boys and 86.9% of girls fall in the long face category (more than 1.699), and the results are statistically significant.

**Table 4:** Tr-Sn/Sn-Gn ratio evaluation with reference to the golden ratio in school-going children Tr-Gn: Trichion-gnathion distance; Sn-Gn: Subnasale-gnathion distance

Tr-Sn/Sn-Gn ratio evaluation with reference to the golden ratio in school-going children						
School-going children with mixed dentition	Male		Female		Total	
	No.	%	No.	%	No.	%
Normal	6	6.4	10	9.4	16	8.0
Long	83	88.3	93	86.9	176	87.6
Short	5	5.3	4	3.7	9	4.4
Total	94	100.0	107	100.0	201	100.0
X² = 0.84, P = 0.66, S						

## Discussion

The qualitative aspects of beauty and facial aesthetics should be quantified to measure and compare with other parameters. One of the ratios used by many authors is the unique ‘divine ratio’ or ‘golden ratio’ or 'golden proportion’. This invincible measurement has been used and compared in the science of aesthetics for years and still holds value to its zenith. There are two schools of thought on the golden ratio. One school believes that the golden ratio is an ideal ratio and the aesthetic parameters should fall in line with it or be near or close to it. The second school believes that there is a lot of variation among varied regions and populations across the globe when it comes to the golden ratio and many authors speculate on the verdict of ideal beauty and its closeness to the divine ratio [8].

The total facial height (TFH) is measured as a linear distance from Tr (trichion: anterior hairline at the midline) to Gn (gnathion: most inferior point on chin). In our study, TFH in boys is more than in girls in both preschool and school-going children of the Santhal tribe in the Bankura district of West Bengal. This finding was significantly associated with sexual dimorphism in preschool children with primary dentition, but no statistically significant value was noted in school-going children with mixed dentition. The result was in line with a study by Kaya et al., wherein a Turkish sample population of 133 individuals aged 18-40 years was analyzed for TFH in association with gender, and a significant correlation of high TFH value in males compared to females was reported [8]. Also, Alam et al. and Packiriswamy et al. inferred in cross-sectional studies of Malaysian subjects that males have larger TFH than females [3,12]. However, on the contrary, Farkas et al. did a study on the Indian population and found that females have more facial height than males [13].

The golden ratio is undoubtedly a unique proportion used to measure beauty in diverse aspects and has been in use for centuries by a plethora of researchers across the globe in varied fields. The field of medicine and dentistry has also applied this divine ratio in different arenas. The studies are in abundance proving the worth and effectiveness of the golden ratio to quantify dental and facial relationships and proportions in aesthetic science [1-3,11,14-16]. A study done using 3D-stereophotogrammetric facial acquisitions of 400 subjects has reported that facial attractiveness cannot be correlated with the golden ratio as seven out of 10 ratios used in their study were statistically different from the divine ratio of 1.618 [17]. It is shown in this research that Tr-Sn/Sn-Gn ratio is lower in males than females in both preschool and school-going children. The mean ratio in boys with primary dentition is 1.93 as compared to 1.97 in girls, and the difference is statistically non-significant. The mean ratio in boys with mixed dentition is 1.86 as compared to 1.90 in girls, but the difference is not statistically significant. Yeung et al., in a study on 12-year-old southern Chinese children, inferred that males have lesser facial height than females and that the lower face height constitutes 56.7% (male) and 55.3% (female) of the total face height. This means that the Sn-Gn is higher in males, leading to a lower Tr-Sn/Sn-Gn ratio when compared to females, which is in accordance with our study. Loveday et al. also reported similar findings in photographic analysis of 200 young adult Igbos from Nigeria, with males displaying a lower third facial height than females. Olusanya et al. in their research also convey similar findings [18-20]. On the other hand, Folaranmi et al. in a study came out with an inference that no significant association exists between gender and lower facial height [21]. In our study, when the Tr-Sn/Sn-Gn ratio was compared against the golden ratio, it was found that the majority of the population falls in the long face category in both preschool and school-going children. This finding is similar to a study by Kaya et al. in Istanbul, wherein 133 Turkish individuals of 18-40 years were studied, and a majority of the population (75.2%), both males and females, were found to fall in the long face category. Only 13.5% were seen to fall in the normal golden ratio category [8]. Lavelle CL conducted a study of male brachycephalic and dolichocephalic populations and inferred that mesiodistal and buccolingual crown diameters of teeth are consistently greater in subjects with short faces than those with long faces [22]. Furthermore, Ferring et al. reported that the disproportionate index to the golden ratio (average percentage of deviation from golden ratio value) in the vertical dimension is more pronounced in both genders as displayed in our study [1]. A recent meta-analysis examining the association of parameters with the golden ratio across different countries inferred that the guide for creating looks and aesthetics varies with the race and ethnicity of the population; this finding aligns with the conclusions of our research [23].

There are a few limitations to our study. As this is the first study of its kind on this young population and the literature is scarce, comparing our outcomes with other research is challenging; therefore, further studies are recommended on the application of the golden ratio in young children. Secondly, while this study sheds light on vertical parameters in reference to the golden ratio, it does not address transverse parameters. Hence, conducting both vertical and transverse facial analysis based on the golden ratio could offer better insights into these prospects of dental sciences.

## Conclusions

The golden ratio is an elusive proportion that a dentist should be aware of. Aesthetic dentistry is the science of looks, balance, and harmony. Therefore, during orthodontic alignment and prosthetic rehabilitation of anterior teeth, the golden ratio should be taken into deliberation. In our study, vertical facial analysis was done and correlated with the golden ratio. We found that children of the Santhal tribe did not conform to the golden ratio, showing long faces in the majority, and only a very small study population was in alignment with the ideal divine proportion. In long face children of both genders, the mesiodistal width and buccolingual width of anterior teeth are less when compared to other facial types and hence this point could be considered while working for the Santhal tribe children.
